# Consultation performance of general practitioners when supported by an asthma/COPDC-service

**DOI:** 10.1186/1756-0500-5-368

**Published:** 2012-07-23

**Authors:** Lucas EM Annelies, Derckx WCC Emmy, Meulepas A Marianne, Smeele JM Ivo, Smeenk WJM Frank, van Schayck P Onno

**Affiliations:** 1 Department of General Practice (HAG), Research Institute Caphri, University Maastricht, PO box 616, Maastricht, MD, 6200, The Netherlands; 2 Stichting Kwaliteit en Ontwikkeling Huisartsenzorg, PO box 2155, Eindhoven, CD, 5600, The Netherlands; 3 Meetpunt Kwaliteit, PO box 6406, EINDHOVEN, HK, 5600, The Netherlands; 4 CAHAG, NHG, Domus Medica, PO box 3231, Utrecht, GE, 3502, The Netherlands; 5 Department of Pulmonary Diseases & Tuberculosis,Catharina Hospital, PO box 1350, Eindhoven, ZA, 5602, The Netherlands

## Abstract

**Background:**

General practitioners (GPs) can refer patients to an asthma/COPD service (AC-service) for diagnostic assessment of spirometry and medical history and for asthma or COPD monitoring. The AC-service reports diagnostic results and additional information about disease burden (BORG-score for complaints, MRC-dyspnoea score, exacerbation rate), life style, medication and compliance, to the patient’s GP. This study explores how GPs use this additional information when discussing the patient’s disease burden and how this influences GPs’ information and education provision during consultations with asthma/COPD patients.

**Method:**

Patients with (a suspicion of) asthma or COPD were referred to an AC-service and consulted their GPs after they had received a report from the AC-service. Retrospectively patients answered questions about their GPs’ performance during these consultations. Performances were compared with performances of the same GPs during consultations without support of the AC-service (usual care), earlier that year.

**Results:**

Of consultations not initiated by an AC-service check-up, 91% focussed on complaints, the initial reason for the consultation. In AC-service supported follow-up consultations, GPs explored disease burden when the (BORG-)score for complaints was high - as reported by the AC-service - even when patients themselves thought it was irrelevant. GPs put significantly less effort in exploring disease burden when the Borg-score was low (BORG 3–4: 69%; BORG1-2: 51%, *p = 0,01*). GPs mostly ignored MRC-dyspnoea scores: attention to dyspnoea was 18% for MRC-score <3 and 25% for MRC-score ≥3 (*p = 0,63).* GPs encouraged physical fitness in 13% of patients. Smoking behaviour was discussed with 66% of the actual smokers but only 14% remembered a stop smoking advice. Furthermore, pharmacotherapeutic management education in AC-service supported consultations did not differ from performance in usual care according to patient evaluations.

**Conclusion:**

Other than taking into account the severity of complaints, there was no difference between GPs’ performance in AC-service supported and in usual care consultations. AC-service reports are thus not effective by themselves. GPs should be encouraged to use the information better and systematically check all relevant aspects that characterize the disease burden of their patients.

## Background

Self management and individual action plans are important issues in effective care for asthma and COPD patients [1-3]. Patient information and education are prerequisites for successful implementation. Through a multifaceted approach, patients should be educated about all aspects of their disease, the influence of lifestyle and medication [4]. They should be supported to implement this knowledge and to adopt a healthier life style. In every day practice this ideal situation is hard to realize. New guidelines focus on the individual burden of the disease for which many factors (*e.g.* respiratory complaints and disabilities, co-morbidity, social restrictions) have to be explored [5]. However, only a limited number of issues can be discussed in one consultation. In addition, addressing lifestyle behaviour is not part of GPs’ routine procedures [6]. Research is needed to determine which management model offers the best support to improve this [7] and to provide good instruments to routinely inform and educate patients.

One of the models is the support of an Asthma/COPD service (AC-service), an efficacious working method in The Netherlands for obtaining best quality diagnosis of asthma/COPD for all patients with respiratory problems in a primary care practice [8]. The service’s main task is to perform diagnostic spirometry on patients referred by their GPs. To improve assessment quality, consulting pulmonologists use structured medical history data in addition to spirometry data. This protocolized approach of the AC-service gives valid diagnostic results [8,9] and provides a lot of additional information to GPs which they can use in consultations with their patients.

### Consultation support by reports of the AC-service

**Figure 1:**
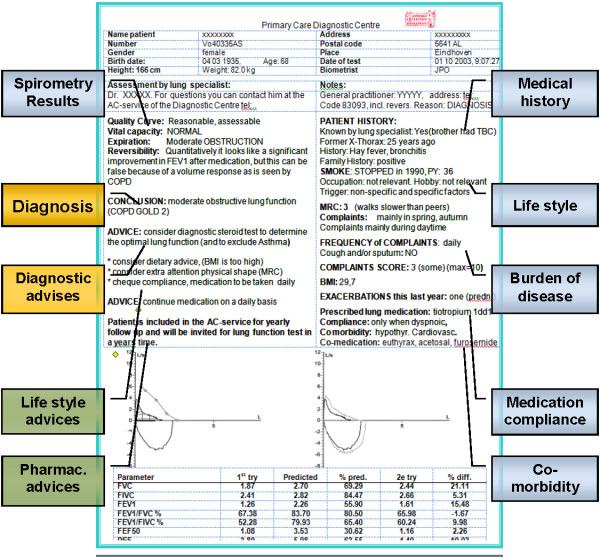
Chapters of information given in the Report of theAC-service.

The AC-service sends diagnostic reports to GPs which include history data. GPs can use this information to structure consultation communication. Communication starts with exploring complaints to understand patient’s disease burden followed by patient-doctor conversations. The AC-service reports a Borg-score (0 – 10) for respiratory complaints, a frequency score for exacerbations per year, the MRC-dyspnoea score (1–5) and weight problems (BMI) (Figure 1). These data can help the GP to discuss disease burden, something not always spontaneously reported by the patient [10] or noticed by the GP [11]. Disease burden might get less attention at follow-up visits that focus on spirometry results even when complaints and scores are substantial.

Tailored recommendations for medical treatment and lifestyle adaptations, *i.e.* stopping smoking and improving physical condition, are also added to the report of the AC-service. Especially when the disease burden indicates the need for therapeutic action these recommendations should be discussed in the next part of the consultation.

Unknown is to what extent GPs use the information of the AC-service in their consultations and whether this influences patients’ knowledge and attitudes towards lifestyle adaptations.

We studied this by exploring the consultation performance of GPs - as perceived by their patients with respiratory problems – when a report of the AC-service was available and compared this with the performance of GPs in usual care consultations.

We focused in detail on the extent to which GPs addressed the experienced burden of the disease (complaints, MRC, exacerbations), the diagnosis, the impact of lifestyle on the disease (smoking, lack of exercise), inhalation technique, and compliance.

## Method

### Setting

This study was part of an extensive research project that evaluated the “Asthma/COPD-service” for its validity and reliability [8,9] and the diagnostic and therapeutic support offered to GPs in their care for patients with respiratory complaints [12-14]. The research project was conducted at the AC-service in Eindhoven, *the Netherlands,* established in 2001 for the purpose of supporting asthma/COPD care in 300 primary care practices in the region. When the research started, AC-service was already part of regular care for some GPs but not all primary care practices in the region were enrolled.

### Participants in the study

From May 2005 until December 2007 GPs from Eindhoven city and surrounding areas who had no experience of working with the AC-service were asked to participate in the research project. This meant that GPs would start with the regular support of the AC-service and agree to have its effect evaluated by their patients. To be able to collect consultation data *without* and *with* support of the AC-service from the same GPs, GPs were asked to postpone the actual partnership with the AC-service for one year after they agreed to participate.

### Regular support of the AC-service

Regular support of the AC-service starts with the screening of primary care office registers. Patients (12 years and older) with respiratory problems are selected who are eligible for diagnostic assessment and/or follow-up in order to enrol in their GP’s monitoring program. Selection criteria are the use of inhaled medication in the last two years (ATC-code R03) and/or a registered diagnosis “COPD” “asthma” or “bronchitis” (ICPC-codes R95, R96 and R91, [15]). GPs can send all these patients - including all new respiratory patients - to the AC-service for written medical history taking and spirometry. GPs decide about the (very) old, immobile, or psychiatric patients for which these referrals not automatically apply.

After each visit to the AC-service, GPs receive a report with the results of spirometry, the medical history and diagnostic and therapeutic advice (Figure 1). The GP remains fully responsible for the interpretation of the report and for the patient’s care process.

### Design

Office registers of participating practices were screened by the AC-service as described. Selected patients were asked to participate in the evaluation of their GP’s care process and to answer questionnaires at the start and at the end of the research period. GPs were informed about which patients were selected only at the end of the research period.

**Figure 2:**
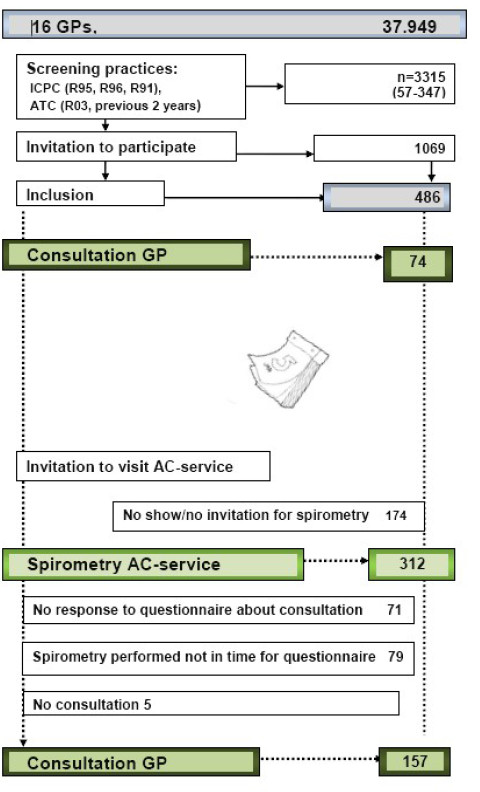
Participating Patients.

At the start of the study data were collected about GPs’ performances in consultations without support of the AC-service (*i.e.* usual care) (Figure 2). Participating patients received a questionnaire eight weeks after their pxractice enrolled in the study. The questionnaire addressed the consultations patients might have had in these eight weeks related to respiratory problems. Patients were asked whether they had discussed the following care issues with their GPs in detail:

· their personal disease burden (complaints, dyspnoea, exacerbations)

· their diagnosis

· the impact of lifestyle on their disease (smoking, lack of exercise)

· medication, inhalation technique and compliance

At the end of the one year research period, GPs asked the selected patients to visit the AC-service as part of their new astma/COPD disease management and to discuss results during consultation.

GPs’ performances during these consultations (supported by a report of the AC-service) were examined with the same questionnaire as used at the start of the study, which was sent to the patients eight weeks after the invitation to visit the AC-service. By that time the actual visit to the AC-service should have taken place, as well as the patient-doctor consultation discussing the results.

In order not to interfere with regular care, no special research protocols were written for neither the AC-service nor the GPs.

Data from usual care consultations were compared to data from consultations supported by information from the AC-service. The influence of this information was examined by taking into account the specific data given to the GP by the AC-service (see Figure 1).

### Data analysis

We compared the frequencies of each issue discussed (means per GP) during usual care and after support of the AC-service (SPSS 17, paired samples test).

The relevance of discussing specific issues could be assessed in AC-service supported consultations by studying impact scores as reported by the AC-service (SPSS 17, *χ*2 test).

### Ethical approval

Participants were offered the usual care common in the local area and in other Dutch regions. At first this was without support of an AC-service. During the research period this support was introduced as part of the new usual care procedure. No ethical approval was needed in this case.

Informed consent was obtained from the participants to analyze the questionnaires as well as the data of the AC-service. Participants answered the questionnaire anonymously.

## Results

### Participating GPs

16 GPs participated in the study. GPs were on average 48 years of age, 30% were female, and 40% of the practices were urban. The total patient population per GP ranged from 1135 to 2987 (Total: 37.946 patients).

### Participating patients

Of 1069 patients selected for the study, 486 agreed to participate (18–63 patients per GP. Mean: 30). In the first 8 weeks of the study 74 patients (2–13 patients per GP) visited their GP for usual care and responded to the questionnaire. At the end of the study, after one year, 321 patients visited the AC-service.

157 (49%) went for a consultation to their GP (6–23 patients per GP) and answered the questions about their GP’s performance. These patients had baseline characteristics comparable to *all* patients visiting the AC-service: COPD prevalence: 17%; ≥1 exacerbation/year: 22%; smoking: 24%: mean MRC-dyspnoea score ≥ 3: 28%; using inhaled corticosteroids: 48%; co-morbidity reported: 17%.

### Exploring the patients’ disease burden

85% of the patients consulting their GP after a visit to the AC-service did not mention complaints as a reason for consultation but 60% had a BORG ≥3 score for complaints. GPs discussed complaints in 77% and exacerbations in 78% of the AC-service supported consultations according to their patients (Table 1). Complaints did get less attention (55%) in telephone consultations (34% of AC-supported consultations).

**Table 1:** Usual care consultations and AC-supported consultations (mean % per GP) in which the burden of the respiratory disease was discussed with the patient

	**No support AC-sItem discussed**	**Support AC-s Item discussed**	**Paired*t*-test means/GP**
Complaints	91%	77%	*p=.75; 95% C.I. -,24/+.34*
Exacerbations	91%	78%	*p=.79; 95% C.I. -.21/+.34.*
Dyspnea total	25%	31%	*p=1,00; 95% C.I. .-26/+.26*
Weight problems total	14%	6%	*p=0,57. 95% C.I. -.24/+.14*

(GPs: n=16. Patients “no ACs-support” n= 5-10/GP, total 74. Patients “ACs-support”: n= 9-21/GP, total 157).

Overall, in consultations without AC-service support (16% were telephone consultations) complaints were discussed more often (mean score GPs: 91%). However, a paired sample test for means per GP showed no significant difference between discussion of complaints with and without AC-services support because some GPs discussed less complaints when supported by the AC-service and others discussed more (*t*_*21*_ *= .32, p = .75. 95% c.i. -.24%/.34*). This was due to differences in complaint severity between patients of different GPs (BORG > 4: ranged 10% - 60% per practice), which was significantly related to the frequency of discussing complaints *(p = 0,01, χ*2*),* (Table 2).

**Table 2:** Mean % of GPs’ consultations in which the burden of the respiratory disease was discussed with the patient, the actual prevalence of the problem as reported by the AC-service and severity weighting for whether or not discussing the item

	**Support AC-s**		
	**Prevalence of item reported by AC-s**	**Item discussed**	***Weighting severity***
BORGscore 0-2^1^	*49%*	51%	***P=0,01***
BORGscore 3	*18%*	69%	
BORGscore ≥ 4	*33%*	90%	
Complaints total	*100%*	77%	
Exacerbations n=0/yr	*78%*	72%	***P=0,04***
Exacerbations n*≥*1/yr	*14%*	90%	
Exacerbations total	*100%*	78%	
MRC-dyspnea 0-1-2	*83%*	29%	***P=0,24***
MRC-dyspnea 3-4-5	*17%*	40%	
Dyspnea total	*100%*	31%	
BMI < 21	*9%*	0%	***P=0,06***
BMI 21-30	*75%*	4%	
BMI 25 -30	*16%*	18%	
Weight problems total	*100%*	6%	

(GPs: n=16. Patients “ACs-support”: n= 9-21/GP, total 157).

BORG-score: respiratory complaints 0-2: no or few complaints 3: complaints; ≥ 4: considerable/many complaints.

Exacerbation: need for prednisolon/antibiotics because of respiratory complaints.

MRC-dyspnea scores: 0= no limitations; 1=dyspnea only when exercising; 2= dyspnea in minor exercise; 3= unable to walk up with people of sameage, same sex, without dyspnea; 4= dyspnea in daily chores; 5= dyspnea in self care.

There was no difference in frequency of discussing dyspnoea (31% of the consultation; *t*_*19*_ *= .00, p = 1,00; 95% C.I. 0,26/+0,26)* between usual care and AC-supported care (Table 2). GPs did not consider the exact MRC-dyspnoea score for discussing dyspnoea (Table 2). 17% of the patients scored ≥3 on the MRC-dyspnoea scale. Of those, 28% said it was not a relevant issue.

GPs paid some attention to overweight problems but not to underweight issues even when this was advised by the AC-service (Table 2)*.* Overall, weight problems only got attention in 6% of the consultations, there was no difference between usual care and AC-supported care (*t*_*18*_ *= .-.56, p = 0,57;. 95% C.I.-.24/+.14).* 50% of the underweight persons thought it was not a health problem as did 25% of the overweight patients.

### Giving information about the diagnosis

After receiving reports from the AC-service, 39% of GPs discussed the diagnosis with their patients. Two thirds of the patients received a “clear explanation” regarding their diagnosis. Only half of them remembered their GP mentioning the name of their diagnosis. Lack of a diagnosis (“no asthma or COPD”) was hardest to explain (“clear information” in 37%). Asthma (70%) and COPD patients (72%) reported to be rather well informed about their diagnosis.

### Lifestyle recommendations

According to the patients, GPs discussed smoking cessation with 66% of smokers after a visit to the AC-service (range 40-100%) and with 71% in usual care (range 50-100%) (Table 3). 14% of smokers in the AC-supported consultations and 19% of smokers in usual care did not recall a smoking advice but answered that the issue was irrelevant. There was no significant difference for remembering stop smoking recommendations (*t*_*-2,3*_ *= .20, p = 0,34; 95% C.I. -.14/+0.05).*

**Table 3:** Lifestyle advice by GPs: mean percentage of consultations in which recommendations for improving lifestyle were given and their relevance according to the report of the AC-service

	**Advice: stop smoking (mean/GP)**					**Advice: improve condition**			
**Available information**	**Actual smoker**	**Stopped smoking**	**Never smoked**	**overall**		**MRC 0-2**	**MRC ≥ 3**	**overall**	
No ACs-support	71%^1^	7%	0%	31%	***P= 0,34***	13%	17%^3^	17%	***P=0,42***
ACs- support	66%^2^	10%	0%	21%		10%	13%^4^	13%	

Overall difference between support and no support (p).

^1,2^ in addition 14%^1^ and 19%^2^ of the smokers said it was irrelevant to discuss stop smoking.

^3,4^ in addition 14%^3^ and 40%^4^ of the patients MRC≥3 said it was irrelevant to discuss how to improve physical shape.

When the MRC-dyspnoea score was provided by the AC-service, GPs advised 13% of the patients with an MRC ≥3 to improve their physical fitness (Table 3). No significant difference between consultations was found for MRC scores and discussing fitness *(t*_*t2,3*_ *= .21 p = 0,42; 95% C.I. -.32/+.14)*

### Managing medication

Patients in usual care and patients with a visit to the AC-service were similarly advised on how they should manage medication (Table 4). At the start of the study 75% of all patients gave themselves 8 points on a 10-point scale for adequately using medication. Only 5% thought they should do better. This figure did not improve.

**Table 4:** Mean % of consultations in which the compliance to prescribed medication and inhaling technique was discussed, instruction was given in inhaling medication and in adapting medication in case of more respiratory problems

**Available information**	**Compliance discussed**	**Inhalation technique discussed**	**Inhalation instruction given**	**Correct dosis/use explained**	**Self management instruction**
No ACs-support	39%	26%	29%	25%	28%
ACs- support	42%	31%	29%	28%	43%
***p***	***(t***_***-2,3***_***=.18,****p=0,18;****95% C.I. -.08/+.40)***	***(t***_***-2,3***_***=.22,****p=0,43;****95% C.I. -.14/+.30)***	***(t***_***-2,3***_***=.20,****p=0,77;****95% C.I. -.28/+.38)***	***(t***_***-2,3***_***=.20,****p=1,0;****95% C.I. -.30/+.30)***	***(t***_***-2,3***_***=.17,****p=0,67;****95% C.I. -.21/+.32)***

Differences between support of the AC-service and no support *(p)*.

## Discussion

It is known that the support of AC-services improves the diagnostic process as well as the logistic organization of asthma/COPD management in primary care [16]. An AC-service’s report does not only provide a diagnosis but also a more complete set of data about a patient’s respiratory condition. We examined whether these reports had a positive influence on the consultation performances of GPs. Our findings, based on patient recall, showed that GPs supported by an asthma/COPD-service did not conduct more comprehensive consultations than GPs who delivered usual care. Van den Bemt found this result for consultations with long time monitored, stable COPD patients [17]. We could not find evidence for her expectation that results should be better for patients recently referred to the AC-service.

AC-service supported consultations were delivered by phone more often than usual care consultations. It is known that telephone consultations are shorter and contain less data-gathering and counselling/advice [18]. This might contribute to but should not be a justification for the lack of improvement, *i.e.* more extensive consultations when more information is available.

A positive finding was that issues discussed were tailored to the scores reported by the AC-service, even when patients had bad scores and thought that this discussion was irrelevant.

In addition, GPs were able to address patients who might not have come for follow up, even if they had complaints, without the invitation to visit the AC-service.

Regular follow up of patients with chronic conditions such as asthma and COPD is recommended by the guidelines. The AC service supports an active follow up system by monitoring asthma and COPD patients upon request of their GPs, thus guaranteeing as much as possible regular follow up independent from complaint presence.

COPD patients in particular are badly informed about their disease [19]. In our study only a minority of the patients could name their diagnosis, and amongst these one third had not clearly understood their GP’s explanation. Patients particularly did not understand their GP’s explanation when the diagnosis asthma or COPD was still uncertain which occurs for 50% of the patients visiting the AC-service for the first time [13]. This stresses the importance for GPs as well as their patients to understand their health problem. Therefore, we recommend to have patients monitored by the AC-service at the very least until their diagnosis is clear before monitoring is decreased or even stopped as suggested by the literature [17,20].

Studying GPs’ performances in detail we found that a stop-smoking advice was adequately given by all GPs independent from the support of the AC-service. Of course it requires a bigger effort to get patients, especially those with COPD [21], to actually stop smoking. Overall, smoking habits in the study population hardly changed: 12% of the smokers stopped in both usual care and ACs-supported practices but 8% of the non-smokers started smoking during the research period.

Advice to improve physical fitness was rarely given. Since one third of the patients with a MRC-dyspnoea score ≥ 3 did not think improving physical fitness was an issue to discuss, GPs should be instructed to pay more attention to the MRC-dyspnoea score as a first step in implementing this important life style item [22].

In usual care and in ACs-supported consultations, GPs gave similar advice on how to manage medication, indicating that triggering by the AC-service was as effective as being triggered by existing complaints. As shown in other research [23] there is much room for improvement. GPs’ awareness should grow but it is probably more effective to delegate patient information and education to nurse practitioners [24] who are known to work in a more structured way [25] and who appreciate protocol support [26].

We realize that diagnostic spirometry results as a reason for consultation could distract GPs and patients from clinical issues. Patients in our extensive research project reported no clinically relevant differences between experienced attention in usual care and in AC-services supported consultations. This part of the study showed that consultations following a visit to an AC-service were at least as complete as consultations in usual care because of complaints.

### Limitations of the study

The subject of our study was consultation behaviour of general practitioners. We could not question the GPs themselves about their care process and the effect of the AC-service support. In order not to interfere with medical routines, we instead chose to question their patients after consultation. Patients questioned at the start and at the end of the study might not have been the same persons. Although GP behaviour cannot be independent from a specific consulting patient, it should be possible to examine general behaviour patterns in GPs consultations with all their patients and the influence of AC-service support on this pattern.

Recall bias was reduced as much as possible through questionnaire timing. Nevertheless, recall bias cannot be excluded entirely because patients’ perceptions about issues being addressed or being important to recall might differ from actual actions taken. However, the final result should be that *the patients* benefit from the support of an AC-service. This justifies using patients’ answers as measures in our study, acknowledging that patients might have to visit their GP more often before all issues are discussed and/or remembered.

In our study, not only the introduction of consultation support to GPs was new but also the invitation for patients to visit the AC-service and their GP’s office. We chose to study the new situation and compare it with the usual one. Through this design, a difference became apparent between patients in usual care and patients consulting their GP after a visit to the AC-service: in usual care patients only named “complaints” as a reason for the encounter while patients that had visited the AC-service mostly mentioned consulting their GP to discuss the results of their spirometry test. They were more likely to have a telephone consultation (69% compared to 93%)

Since complaints and face-to-face visits are likely to trigger GPs to inform and educate patients more comprehensively, we are pleased to find that there was no difference between usual care (complaints) and AC-supported care (often no complaints). We might have found a more positive influence of the AC-service report if we had compared follow-up consultations fully supported by the AC-service and follow-ups supported only by spirometry results, excluding telephone consultations. Both situations were artificial, and therefore were not chosen for this real life study.

## Conclusion

An AC-service can support GPs in structuring communication with their patients about many aspects of their disease but no significant communication differences were found for consultations supported by AC-service reports compared to usual care consultations.

GPs should be encouraged to use the information and advice given by the AC service and to discuss this with their patients in a structured manner. Awareness about the full content of the report should be given sufficient attention by all Asthma/COPD services supporting GPs.

## Competing interest

The authors declare that they had no competing interest.

## Author’s contribution

AEML, IJMS, FEJMS and CPvS conceived of the study and set up its design. AEML coordinated the data collection, its statistical analyses and performed the interpretation of the results, supported by EWCCD, AMM, IJMS, FEJMS and CPvS. AEML drafted the manuscript, all authors contributed to the writing and read and approved the final manuscript.
